# Supplementary material for: Modeling glioblastoma heterogeneity as a dynamic network of cell states
Source: Mol Syst Biol. 2021 Sep 16;17(9):e10105. doi: 10.15252/msb.202010105 (PMC8444284; doi:10.15252/msb.202010105)
Supplement: Supplementary file 5 — Source Data for Figure 3 [file MSB-17-e10105-s001.zip › Figure3A_sourcedata/GSEA_3065/hallmarks_state4.GseaPreranked.1623416296068/HALLMARK_APOPTOSIS.html]

Details for gene set HALLMARK\_APOPTOSIS[GSEA]

|  || Dataset | state4 |
| Phenotype | NoPhenotypeAvailable |
| Upregulated in class | na\_neg |
| GeneSet | HALLMARK\_APOPTOSIS |
| Enrichment Score (ES) | -0.41483787 |
| Normalized Enrichment Score (NES) | -1.4526103 |
| Nominal p-value | 0.024930747 |
| FDR q-value | 0.075022146 |
| FWER p-Value | 0.663 |
Table: GSEA Results Summary

  

Fig 1: Enrichment plot: HALLMARK\_APOPTOSIS      
 Profile of the Running ES Score & Positions of GeneSet Members on the Rank Ordered List

  

| PROBE | GENE SYMBOL | GENE\_TITLE | RANK IN GENE LIST | RANK METRIC SCORE | RUNNING ES | CORE ENRICHMENT || 1 | SAT1 |  |  | 4 | 0.669 | 0.0533 | No |
| 2 | FEZ1 |  |  | 42 | 0.313 | 0.0725 | No |
| 3 | BNIP3L |  |  | 54 | 0.294 | 0.0945 | No |
| 4 | LGALS3 |  |  | 89 | 0.259 | 0.1098 | No |
| 5 | TXNIP |  |  | 92 | 0.254 | 0.1300 | No |
| 6 | CLU |  |  | 95 | 0.252 | 0.1500 | No |
| 7 | PEA15 |  |  | 112 | 0.243 | 0.1670 | No |
| 8 | HSPB1 |  |  | 180 | 0.211 | 0.1732 | No |
| 9 | GPX4 |  |  | 183 | 0.210 | 0.1898 | No |
| 10 | CDKN1A |  |  | 319 | 0.171 | 0.1818 | No |
| 11 | SQSTM1 |  |  | 390 | 0.155 | 0.1830 | No |
| 12 | SOD1 |  |  | 396 | 0.152 | 0.1945 | No |
| 13 | BAX |  |  | 521 | 0.134 | 0.1852 | No |
| 14 | TGFB2 |  |  | 537 | 0.132 | 0.1934 | No |
| 15 | IFITM3 |  |  | 546 | 0.132 | 0.2027 | No |
| 16 | DAP |  |  | 637 | 0.121 | 0.1980 | No |
| 17 | BTG2 |  |  | 689 | 0.116 | 0.1990 | No |
| 18 | VDAC2 |  |  | 795 | 0.104 | 0.1905 | No |
| 19 | PDCD4 |  |  | 796 | 0.104 | 0.1989 | No |
| 20 | MMP2 |  |  | 813 | 0.103 | 0.2045 | No |
| 21 | ADD1 |  |  | 829 | 0.101 | 0.2103 | No |
| 22 | TIMP2 |  |  | 1194 | 0.076 | 0.1576 | No |
| 23 | TSPO |  |  | 1302 | 0.071 | 0.1460 | No |
| 24 | SPTAN1 |  |  | 1371 | 0.067 | 0.1404 | No |
| 25 | CDKN1B |  |  | 1650 | 0.057 | 0.1001 | No |
| 26 | RELA |  |  | 1693 | 0.055 | 0.0977 | No |
| 27 | GSN |  |  | 1935 | 0.049 | 0.0627 | No |
| 28 | SATB1 |  |  | 2091 | 0.044 | 0.0412 | No |
| 29 | CREBBP |  |  | 2250 | 0.040 | 0.0189 | No |
| 30 | DPYD |  |  | 2307 | 0.039 | 0.0129 | No |
| 31 | CCND2 |  |  | 2367 | 0.038 | 0.0064 | No |
| 32 | DAP3 |  |  | 2419 | 0.037 | 0.0012 | No |
| 33 | MCL1 |  |  | 2464 | 0.036 | -0.0031 | No |
| 34 | NEDD9 |  |  | 2524 | 0.034 | -0.0099 | No |
| 35 | CD44 |  |  | 2687 | 0.031 | -0.0336 | No |
| 36 | CASP6 |  |  | 2748 | 0.030 | -0.0409 | No |
| 37 | RHOB |  |  | 2911 | 0.026 | -0.0650 | No |
| 38 | PPP2R5B |  |  | 2925 | 0.026 | -0.0650 | No |
| 39 | RARA |  |  | 2942 | 0.026 | -0.0655 | No |
| 40 | CYLD |  |  | 3005 | 0.025 | -0.0735 | No |
| 41 | XIAP |  |  | 3272 | 0.019 | -0.1150 | No |
| 42 | CTNNB1 |  |  | 3329 | 0.017 | -0.1226 | No |
| 43 | RETSAT |  |  | 3588 | 0.009 | -0.1636 | No |
| 44 | ERBB2 |  |  | 3828 | -0.000 | -0.2023 | No |
| 45 | BID |  |  | 3898 | -0.003 | -0.2131 | No |
| 46 | ROCK1 |  |  | 3943 | -0.006 | -0.2198 | No |
| 47 | JUN |  |  | 3979 | -0.008 | -0.2248 | No |
| 48 | CFLAR |  |  | 4051 | -0.012 | -0.2352 | No |
| 49 | PSEN1 |  |  | 4117 | -0.017 | -0.2444 | No |
| 50 | ATF3 |  |  | 4262 | -0.026 | -0.2656 | No |
| 51 | DNM1L |  |  | 4271 | -0.027 | -0.2648 | No |
| 52 | ISG20 |  |  | 4361 | -0.033 | -0.2765 | No |
| 53 | F2R |  |  | 4370 | -0.033 | -0.2751 | No |
| 54 | BMP2 |  |  | 4398 | -0.036 | -0.2766 | No |
| 55 | PPT1 |  |  | 4417 | -0.037 | -0.2766 | No |
| 56 | ANKH |  |  | 4600 | -0.051 | -0.3019 | No |
| 57 | DNAJA1 |  |  | 4635 | -0.054 | -0.3030 | No |
| 58 | IGF2R |  |  | 4653 | -0.056 | -0.3013 | No |
| 59 | ERBB3 |  |  | 4720 | -0.061 | -0.3070 | No |
| 60 | CDK2 |  |  | 5015 | -0.084 | -0.3478 | No |
| 61 | IRF1 |  |  | 5157 | -0.098 | -0.3627 | No |
| 62 | APP |  |  | 5192 | -0.101 | -0.3601 | No |
| 63 | RHOT2 |  |  | 5253 | -0.106 | -0.3613 | No |
| 64 | GADD45A |  |  | 5297 | -0.109 | -0.3594 | No |
| 65 | BCAP31 |  |  | 5477 | -0.129 | -0.3780 | No |
| 66 | ANXA1 |  |  | 5538 | -0.135 | -0.3768 | No |
| 67 | DNAJC3 |  |  | 5774 | -0.178 | -0.4005 | Yes |
| 68 | CAV1 |  |  | 5842 | -0.193 | -0.3957 | Yes |
| 69 | PMAIP1 |  |  | 5871 | -0.199 | -0.3841 | Yes |
| 70 | BTG3 |  |  | 5895 | -0.206 | -0.3713 | Yes |
| 71 | WEE1 |  |  | 5907 | -0.209 | -0.3562 | Yes |
| 72 | PLAT |  |  | 6079 | -0.285 | -0.3609 | Yes |
| 73 | LMNA |  |  | 6105 | -0.300 | -0.3408 | Yes |
| 74 | SLC20A1 |  |  | 6112 | -0.306 | -0.3170 | Yes |
| 75 | EBP |  |  | 6133 | -0.324 | -0.2941 | Yes |
| 76 | EMP1 |  |  | 6166 | -0.357 | -0.2705 | Yes |
| 77 | TIMP1 |  |  | 6191 | -0.409 | -0.2414 | Yes |
| 78 | BRCA1 |  |  | 6197 | -0.435 | -0.2071 | Yes |
| 79 | CCND1 |  |  | 6203 | -0.454 | -0.1713 | Yes |
| 80 | TNFRSF12A |  |  | 6251 | -0.658 | -0.1258 | Yes |
| 81 | HMGB2 |  |  | 6258 | -0.766 | -0.0650 | Yes |
| 82 | TOP2A |  |  | 6260 | -0.814 | 0.0005 | Yes |
Table: GSEA details [plain text format]

  

Fig 2: HALLMARK\_APOPTOSIS: Random ES distribution      
 Gene set null distribution of ES for **HALLMARK\_APOPTOSIS**
